# Velamentous insertion of umbilical cord with vasa praevia: case series and literature review

**Published:** 2016

**Authors:** RE Bohîlțea, MM Cîrstoiu, AI Ciuvica, O Munteanu, O Bodean, D Voicu, CA Ionescu

**Affiliations:** * ”Carol Davila” University of Medicine and Pharmacy, Bucharest, Romania; ** “Alfred Rusescu” Institute for Mother and Child Care, Bucharest, Romania; *** University Emergency Hospital Bucharest, Romania; **** “Sf. Pantelimon” Clinical Emergency Hospital, Bucharest, Romania

**Keywords:** vasa praevia, velamentous insertion, ultrasound cervical assessment

## Abstract

A velamentous umbilical cord is characterized by membranous umbilical vessels at the placental insertion site that are prone to compression and rupture, especially when they are located in the membranes covering the cervical os (vasa praevia). The velamentous insertion of the umbilical cord, with a reported incidence of 1% in singleton pregnancies and 15% in monochorionic twin gestations, has been associated with obstetric complications: fetal growth restriction, prematurity, congenital anomalies, low Apgar scores, fetal bleeding with acute fetal distress and placental retention. The pathogenesis is unknown, but the trophotropism theory is the most common and supported by the association of velamentous cord insertion and placenta praevia. The prevalence of vasa praevia is of approximately 1/ 2500 deliveries; the risk factors include the use of assisted reproductive technologies, low-lying placenta or placenta praevia, bilobed or succenturiate lobe placenta and multiple gestation. The diagnosis is rarely established before delivery and consequently the fetal mortality is extremely high. We report two cases of velamentous marginal umbilical cord insertion associated with vasa praevia (type 1 vasa praevia) and placenta praevia diagnosed during a routine mid-trimester fetal 2D ultrasound scan, color and power Doppler transvaginal ultrasound cervical assessment. The ultrasound examination revealed one umbilical vessel crossing the internal os of the cervix entering the placental margin and connecting to the subchorionic vasculature, remaining immobile when the uterus was shaken, the color Doppler imaging enhancing the identification of the vessel. The patients were admitted to the hospital in the third trimester and deliveries were planed and successfully performed at 38 weeks gestation, being confirmed by a macroscopic examination ultrasound diagnostic.

## Introduction

The velamentous insertion presupposes an intermembranous pathway of the umbilical vessels and can be located on the placental area, as aberrant ramifications of a marginal insertion, interlobular in the case of a bilobed placenta or in the case of the presence of the accessory placental lobe. The lack of protection of Wharton jelly raises the risk of compression and rupture, especially in the case of vasa praevia. The prevalence of the velamentous insertion is of 1% in singleton pregnancies [**1**], 15% in monochorionic twin gestations [**2**], while the prevalence of vasa praevia is of approximately 1/ 2500 deliveries [**3**]. In 90-95% of the cases, vasa praevia is associated with praevia/ low-lying, bilobed, or succenturiate lobe placenta or with the velamentous marginal umbilical cord insertion [**4**,**5**]. 

The etiopathogeny is still unknown, but the trophotropism theory, which was launched in 1980 by Kouyoumdjian is plausible and affirms that the focal placental development and atrophy are dependent on factors that determine the relative myometrial perfusion, the insertion of the umbilical cord modifying its initial position according to the placental pole migrating towards the more vascularized uterine area [**6**]. 

The main risk factors are the use of assisted human reproduction techniques, which increase the prevalence of velamentous insertion and of vasa praevia 10 times, multiparous pregnancy, placental anomalies, such as low-lying, placenta praevia, placenta with an accessory lobe or bilobed placenta, and placenta with the umbilical cord that has a single umbilical artery [**7**]. 

The velamentous insertion, which is especially accompanied by vasa praevia, has been associated with a high incidence of fetal growth restriction, prematurity, fetal anomalies, acute fetal distress, and placental retention. The compression exerted can determine fetal hypoxia, vascular thrombosis, and, the rupturing of the vessels, which usually accompany the rupture of the membranes, leads to fetal bleeding and death, which may rapidly occur. However, two trials enrolling 1000 cases of marginal and velamentous insertions confirmed a light increase in the incidence of fetal complications only for the cases of velamentous insertion [**8**,**9**]. The most extensive study regarding the anomalies of the umbilical cord insertion was a Norwegian population study summing 11 000 cases, published in 2015. It reported a statistically significant increase of hemorrhage and obstetrical maneuvers in the third stage of labor only for the velamentous insertion of the umbilical cord [**10**].

The prenatal diagnosis represents the key element of fetal prognosis. The real-time color Doppler transvaginal ultrasound examination highlights the umbilical vessel pathway, which crosses the internal os or passes at less than 2 cm from it with a variable sensitivity of 53-100% [**11**]. The vasa praevia screening of the high-risk patients proved to be cost-efficient [**12**]. 

The most reliable obstetrical approach of vasa praevia cases is represented by the performance of the C-section before the beginning of the labor. The membranes rupture, the repetitive and variable decelerations resistant to tocolysis, the beginning of labor, or the vaginal hemorrhage with fetal blood or accompanied by tachycardia or an irregular pattern of the fetal heartbeats require an emergency C-section [**13**]. The recommendations regarding the antepartum monitoring are various and consist in the admission of the patients in a third level center starting with the 30-32 weeks of gestation age, a prophylactic administration of corticosteroids during the period of 28-32 weeks of gestation and the performing of the non-stress test with a frequency that ranges between twice per week and of 2-3 times/ day [**13**,**14**]. The C-section for vasa praevia can be planned in the period of 34-37 weeks of gestation age [**15**,**16**]. 

## Case report

We reported two cases of marginal velamentous insertion of vasa praevia umbilical cord (type I vasa praevia), associated with the praevia localization of the placenta, diagnosed while performing a routine transvaginal ultrasound scan of the cervix by using a 2D ultrasound, power, color and HD-Flow Doppler imaging, performed during the second trimester ultrasound screening for fetal anomalies. 

Case 1: Caucasian female, aged 30, secundiparous after a vaginal birth, pregnancies obtained spontaneously, without any medical history of abortions, was diagnosed with low-lying inserted placenta and velamentous cord insertion type I vasa praevia [**13**] at 23 weeks + 2 days gestational age. She was admitted for observation in “Polizu” Obstetrics and Gynecology Clinic in the 3rd trimester of gestation, giving birth to a normoponderal female child through an elective C-section without complications at 38 weeks.

Case 2: Caucasian female, aged 35, primiparous with a pregnancy obtained in the absence of using the assisted human reproduction techniques, was diagnosed with the same placenta pathology at 21 weeks and 4 days of gestation. She was admitted in the University Emergency Hospital Bucharest for painful uterine contractions at 27 weeks of gestation, delivering an alive female fetus, of 3250g, with an APGAR score of 9, through a C-section for an uncertain fetus status at 37 weeks and 4 days. The C-section was performed without any intraoperative complications. 

Both patients have benefited from the prophylactic administration of dexamethasone according to the standard scheme of treatment with the purpose of accelerated fetal pulmonary maturity. The antepartum monitorization which took place in the University Emergency Hospital Bucharest consisted in a non-stress test twice per week and a weekly ultrasound examination including the transvaginal evaluation of the cervical length, especially looking for the semiquantitative estimation of the amniotic fluid, the length of the cervical canal, the motility degree of the presentation and the cerebral-placental ratio. Tocolysis consisting of 400mg/ day natural micro dosed progesterone was administered intravaginally during the whole hospitalization period. 

**Fig. 1:**
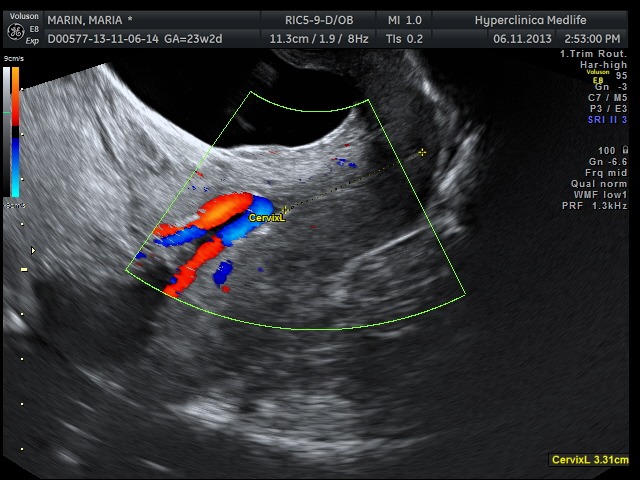
Case 1: measurement of the cervical length by color Doppler transvaginal examination; vessel with a fixed position, which crosses the internal cervical os

**Fig. 2:**
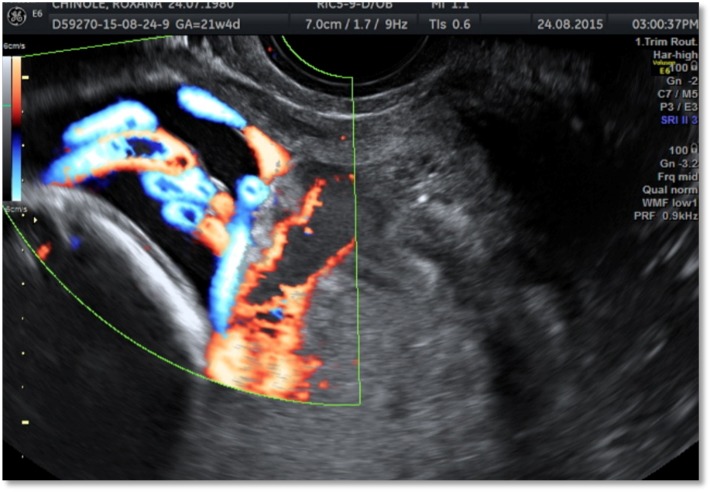
Case 2: HD flow transvaginal Doppler examination; velamentous insertion of the low-lying placenta with vasa praevia

Only while using the Doppler function, in both cases the transvaginal ultrasound scan performed in order to determine the cervical length during the second trimester ultrasound screening for fetal anomalies has showed the presence of an umbilical vessel with a fixed position during the movement of the cervix, crossing the internal cervical os in its way to the placenta margin, where the velamentous insertion continued with the subchorionic vascularization (**Fig. 1**,**2**). 

**Fig. 3:**
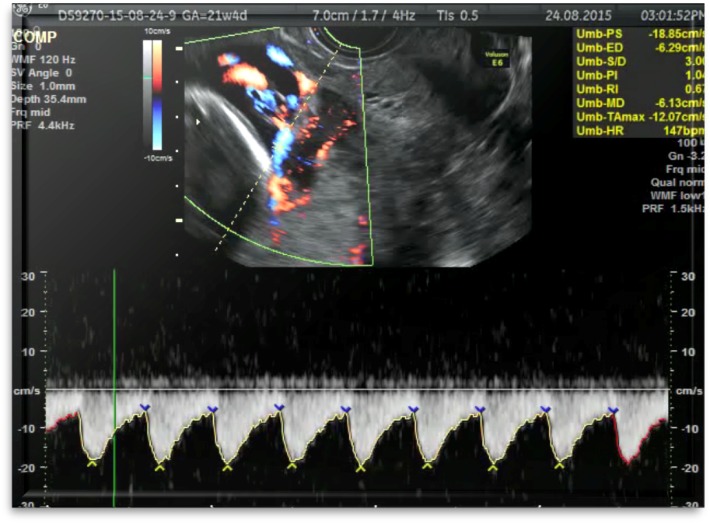
Case 2: pulsated Doppler transvaginal examination: Doppler spectrum characteristic for the vasa praevia umbilical artery

The pulsated Doppler examination identified the vessel as being the umbilical artery (**Fig. 3**). 

**Fig. 4:**
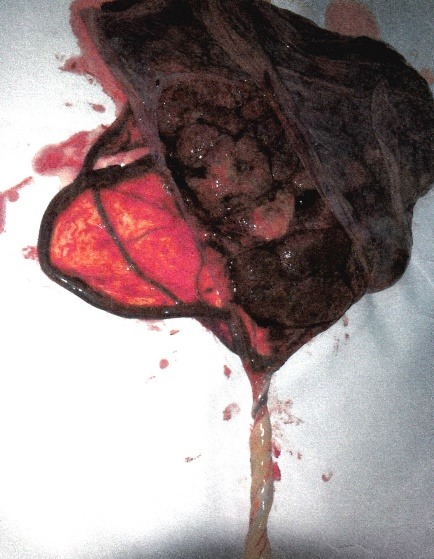
Case 1: macroscopic image of the placenta with a velamentous insertion of the umbilical cord and vasa praevia perfectly collected

**Fig. 5:**
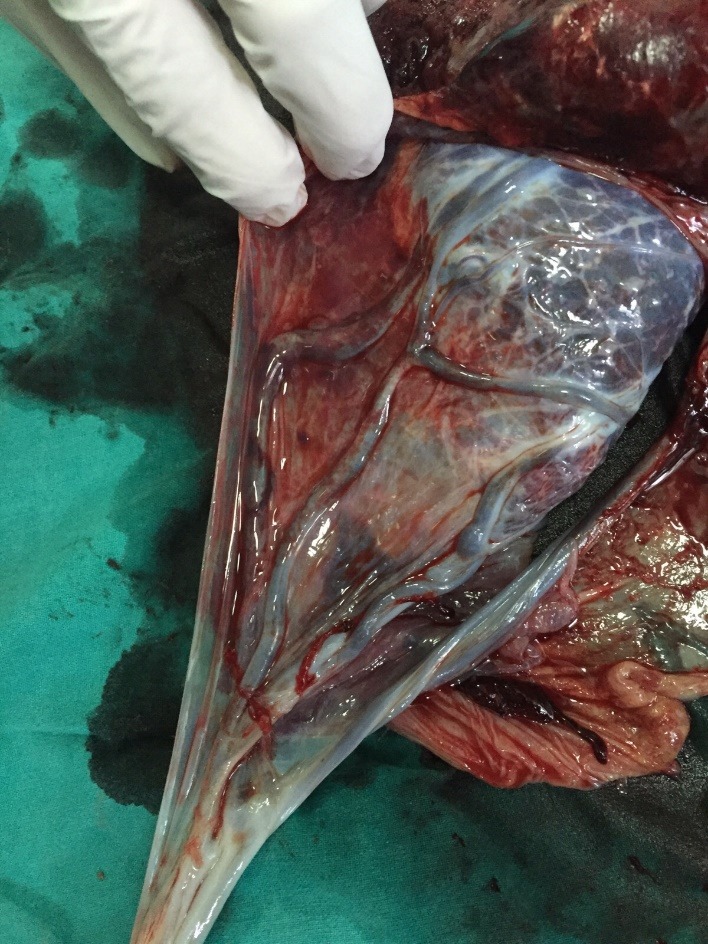
Case 2: macroscopic image of the placenta with a velamentous insertion of the umbilical cord

The macroscopic examination of the placentas confirmed the ultrasound diagnosis (**Fig. 4**,**5**)

## Discussions

The role of these case presentations is to draw the attention of the obstetrical practitioners to the importance of the detailed ultrasound examination of the fetal annexes and especially to the role of transvaginal examination of the cervix, given the simplicity of transvaginal measurement, the raised compliance of the patients, but particularly the proved efficiency of prevention of accidents caused by vasa praevia rupture. The universal screening by transvaginal measurement of the cervix is not routinely recommended by the 2013 Cochrane review due to the lack of evidence and was not currently adopted by the specialty forums. The guidelines of the International Society of Ultrasound and Gynecology (ISUOG) for the second trimester examinations mention the position of the placenta: “The placental location, its relationship with the internal cervical os and its appearance should be described. Examples of abnormal placental findings include the presence of hemorrhage, multiple cysts with triploidy and placental masses such as chorioangioma. In most cases of the routine second-trimester examination, transabdominal ultrasonography permits a clear definition of the relationship between the placenta and the internal cervical os. If the lower placental edge reaches or overlaps the internal os, a follow-up examination in the third trimester is recommended”. What seems to be the most important issues are the evaluation of the scar post C-section, the optional emphasis of the 3 vessels, the examination of the abdominal insertion of the umbilical cord, whose role is to exclude the defects of the anterior abdominal wall, however, the examination of the placenta insertion is not mentioned, the Doppler examination is not routinely recommended, and the measurement of the cervical length is not accepted as a screening method [**17**]. The Romanian Society of Ultrasound in Obstetrics and Gynecology (RSUOG) recommends the optional transvaginal measurement of the cervix and, for the second trimester, it mentions the necessity of evaluating the abdominal insertion of the umbilical cord, highlighting the paravesical umbilical arteries, placenta insertion and the pathology of the umbilical cord whenever possible.

Our approach regarding the patients without risk factors consists in the transvaginal measurement of the cervix in standard conditions, during each prenatal consultation until the 32nd week of gestation, when the ultrasound examination for third trimester fetal anomalies is performed [**18**]. The evaluation of the placental insertion during the second trimester screening ultrasound is very important due to the cases presented, the use of Doppler function being also a routine examination in detecting the placental insertion of the umbilical cord.

In our opinion, the transvaginal evaluation of the cervix should be included in the second trimester screening ultrasound, which represents a screening stage of vasa praevia for premature birth, in order to correctly establish the relationship between the low-lying inferior placenta and the internal cervical os, as well as the evaluation of the cervical arteries. Moreover, we consider the objectification of the placental insertion of the umbilical cord under Doppler examination essential in pregnancies with a high risk of velamentous insertion and vasa praevia: placenta praevia, low-lying placenta, bilobed or accessory lobe placenta, multiple gestation and gestation obtained through assisted human reproduction techniques. 

In conclusion, given the fact that prenatal diagnosis of the placental anomalies and of the umbilical cord decreases the incidence of emergency C-section and of intrauterine fetal death with over 50%, the ultrasound exploratory demands should be increased by including rigid criteria addressed to the specific risk categories of patients. In the context in which the safety of giving birth to a child is a major objective of prenatal examination, both transvaginal and Doppler ultrasound examination of the cervix and umbilical cord placental insertion represent key elements for diagnosis of life threatening conditions. 
